# Association between Cardiovascular Mortality and Economic Development: A Spatio-Temporal Study for Prefectures in Japan

**DOI:** 10.3390/ijerph17041311

**Published:** 2020-02-18

**Authors:** Emerson Augusto Baptista, Kaoru Kakinuma, Bernardo Lanza Queiroz

**Affiliations:** 1 Asian Demographic Research Institute (ADRI), Shanghai University, Shanghai 200444, China; 2 Frontier Research Institute for Interdisciplinary Sciences, Tohoku University, Sendai 980-8578, Japan; 3 Department of Demography, Universidade Federal de Minas Gerais (UFMG), Belo Horizonte 31270-901, Brazil

**Keywords:** mortality, cardiovascular mortality, gross domestic product (GDP), economic development, bivariate choropleth map, Japan, prefectures, Global Burden of Disease Study 2017

## Abstract

In this paper, we use a bivariate choropleth map to investigate the relationship between mortality from cardiovascular disease (CVD) and gross domestic product (GDP) per capita, by sex, in Japanese prefectures from 1996 to 2015. The overall results show a decline in age-standardized CVD mortality rates in all prefectures, for both men and women, and suggest that GDP per capita has varied over the period. We also observed that the relationship between CVD mortality rates and GDP per capita at the prefecture level does not have an overall pattern of the same or inverse association, but is instead a heterogeneous relationship. We argue that this study provides useful clues to policy makers for establishing effective measures for public health planning and the prevention of deaths from CVD. As demonstrated by this study, mapping of the CVD burden in Japan helps to clarify regional differences in life expectancy and health status across regions and identify prefectures where more targeted policy attention may be needed.

## 1. Introduction

Non-communicable diseases (NCDs) accounted for approximately 73% of all deaths worldwide in 2017 [1]. Among the deaths from NCDs, the leading causes are cardiovascular diseases (CVDs) [2,3,4]. In the last 27 years of available data, the estimated number of people who die from CVDs annually increased from approximately 12.0 million in 1990 to 17.8 million in 2017. These numbers represent around 25.6% and 31.8% of all global deaths, respectively [1]. This increase is attributable to a combination of several factors, such as demographic (e.g., driven by population growth and aging), socioeconomic (people with lower incomes have restricted access to quality health services and limited resources to prevent disease), environmental (the exposure to poor air quality—high average concentrations of PM2.5—over the course of several years), behavioral (exposure to tobacco smoking, a sedentary lifestyle and a diet rich in salt intake, sugar, and fat), and epidemiological (improvement in health conditions of the population, access to health services and in premature diagnoses, which increases life expectancy) [5,6,7,8,9,10,11,12,13,14,15,16].

According to the World Health Organization [4] and Franco et al. [17], over three quarters of deaths from CVD take place in low- and middle-income countries, where exposure to risk factors associated with CVD mortality still persists and continues to be a primary focus of public-health endeavors. On the other hand, in high-income countries, where the decline in CVD mortality was substantial over the past half-century, there is recent evidence to suggest that the long-term decline in CVD mortality may be stagnating, and in some countries rates may be even rising [1,16], such as Japan.

In the 20th century, Japan successfully achieved an improvement in the health status of its population, which caught the attention of the rest of the world [18]. Nevertheless, the country observed an increase in the CVD mortality rate from 227 per 100,000 in 1990 to 286.76 in 2017. In the same period, the number of deaths from cardiovascular disease jumped from 285,701.13 to 368,091.30, while the percentage (relative to total number of deaths) dropped from 34.90% to 26.83%, although a slowdown in this decline has been observed in recent years [1]. CVD mortality ceased to be the leading cause of death in the country in 1996, behind deaths from neoplasms (the percentage of deaths from neoplasms increased from 28.47% in 1990 to 30.23% in 2017). Even so, CVD remains one of the main causes of deaths in Japan, although the level, pace of progress, and rates are not uniformly distributed within the country [19]. Regional disparities in the distribution of health-disease patterns among countries are very important [12,13,20,21,22]. Regardless of the level of development, stage of the epidemiological transition, or age composition, the fact is that the geographical variation in cardiovascular mortality is not uniform within the country [3,14,23].

Due to heterogeneity in the spatial distribution of deaths from cardiovascular diseases [19], the goal of this paper is to investigate the relationship between CVD mortality and economic development, measured by gross domestic product (GDP) per capita, in the adult population (over 30 years of age), by sex, in Japanese prefectures from 1996 to 2015. Income, often expressed as gross domestic product (GDP) per capita, is one of the most widely used socioeconomic predictors of mortality/health, and this relationship has been widely discussed in the literature [24,25,26,27,28,29]. We use an adapted bivariate choropleth map and bubble charts using R software to investigate temporal and spatial relationships between CVD mortality and GDP per capita in the country.

## 2. Materials and Methods

### 2.1. Data Sources

We use data on age-specific (population over 30 years and in 5-year age groups up to 95 years or more), sex-specific, and cause-specific mortality from cardiovascular disease (rheumatic heart disease, ischemic heart disease, stroke, hypertensive heart disease, non-rheumatic valvular heart disease, cardiomyopathy and myocarditis, atrial fibrillation and flutter, aortic aneurysm, peripheral artery disease, endocarditis, other cardiovascular and circulatory diseases) from the Global Burden of Disease Study 2017 [1], coordinated by the Institute for Health Metrics and Evaluation (IHME) and publicly available online (http://www.healthdata.org/).

The Global Burden of Disease study was created to provide comprehensive and comparable global health metrics. Estimates of cause-specific mortality, burden of diseases, injuries, and risk factors are reported by year (1990–2017), location (195 countries and territories, and at the subnational level for a subset of countries), age, and sex. GBD also supplied the population estimates used in this paper. Data sources used to produce these estimates came from 1257 census and 761 population registry location-years.

Finally, GDP data by prefecture were extracted from the Cabinet Office, Government of Japan, and are also publicly available. We used two series of annual GDP datasets (1996–2009 and 2001–2015). Both datasets are calculated by using the 1993 System of National Accounts (SNA), which is an international standard index managed by the UN for calculation of GDP. The benchmark year is 2000 for 1996–2009 and 2005 for 2001–2015, and values are given in Yen (¥).

### 2.2. Level of Analysis

**Figure 1:**
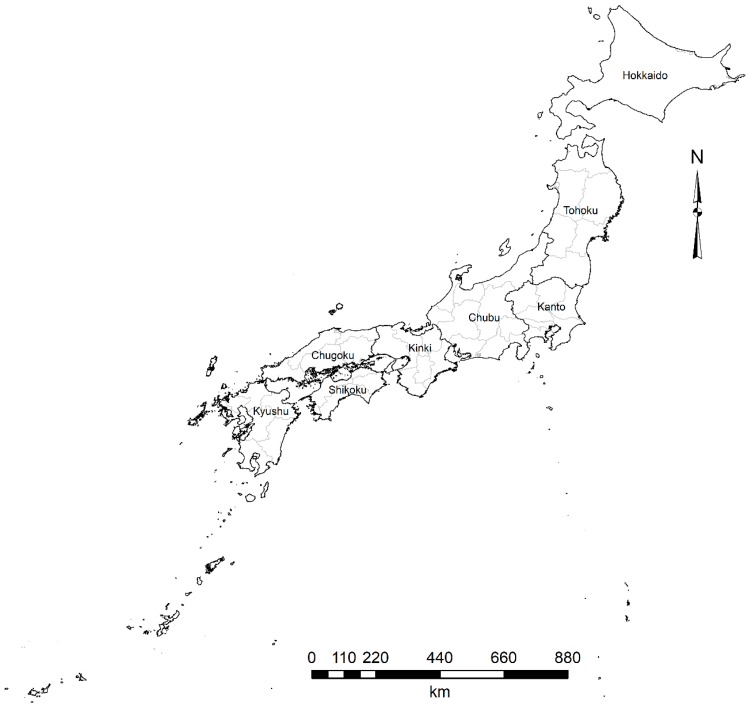
Japan by regions and prefectures.

The units of analysis were the 47 Japanese prefectures, divided into eight regions (Hokkaido, Tohoku, Kanto, Chubu, Kinki, Chugoku, Shikoku, and Kyushu) (Figure 1).

For purposes of analysis, and in order to adjust the annual fluctuations that may occur, we used four time series 1996–2000, 2001–2005, 2006–2010, and 2011–2015. Deaths from cardiovascular disease and population were organized by age (in 5-year age groups up to 95 years or more) and sex. We then calculated sex-specific age-standardized death rates per 100,000 for each prefecture using the Japanese population in 2010 as the standard.

### 2.3. Bivariate Maps

A univariate choropleth map uses colors that portray the spatial variation of a single attribute in the geographic region under study. Migration rate by census tract, excess risk of disease by municipality, level of education by state, and Human Development Index (HDI) by country are some examples. Bivariate choropleth maps follow the same concept, except for displaying two variables simultaneously using a single-color scheme [30]. In fact, bivariate maps go further and allow us to estimate the degree or spatial pattern of cross-correlation between variables, something, to our knowledge, not yet explored in health, epidemiological, and demographic studies. The resultant bivariate map provides useful information for public health in order to identify local areas for resource allocation decisions or health interventions. One additional advantage is that it allows one to quickly identify target areas.

We used an adapted bivariate choropleth map based on Grossenbacher and Zehr’s tutorial [31] in R to evaluate the degree of relation between mortality by cardiovascular disease and GDP per capita at the prefecture level. To match the nine different colors with appropriate classes, we calculated 1/3-quantiles for both variables. Then, the prefectures were put into the appropriate class corresponding to their average CVD and GDP per capita. In addition to bivariate maps, we presented descriptive statistics for the main results over time and across regions. We also tested for the correlation of the variables across time and regions. 

We complemented the bivariate choropleth maps analysis with bubble charts. They added a temporal perspective to the data, that is, they allowed us to visualize the evolution of the relationship studied over the years. The bubble charts were created in R using the plotly package and, in addition to the two variables studied, present the population of each prefecture as a third dimension. Data is publicly available, but we provide full access to all data and routines used in this paper on GitHub.

## 3. Results

From 1990 to 2015, life expectancy in Japan increased by 4.2 years. However, there was an increase in the gap between lowest and highest life expectancy across prefectures. In 1990 the difference was 2.5 and increased to 3.1 in 2015. In addition, the rates of decline in overall mortality presented greater variation across regions over time [19]. Nomura et al. [19] show that Okinawa (3.2) showed the smallest increase whereas Saga (4.8) the largest increase in life expectancy. All-cause mortality rates also declined rapidly from 1990 to 2015, approximately 29%, and large regional variations were observed during the period. The slowest decline was observed in Okinawa and the fastest decline in Shiga [19]. Their results also indicate that overall mortality levels present very weak correlation with health expenditures per capita.

Table 1 presents the mean, standard deviation, *t*-test, *p*-value (ANOVA), and 95% confidence interval for CVD mortality rates and GDP per capita across regions, by sex and over the periods under analysis. The Tohoku and Kanto regions have a higher average CMRCVD than that the country (overall) for both men and women. In the case of women, there is still the Kinki region. The mean GDP per capita is higher than the country in the regions of Kanto, Chubu, and Kinki. While CVD mortality rates have declined for both men and women in all regions, the income differential has not changed much over the 20 years. In addition, in the analysis, the relationships between the last period (2011–2015) and the others were statistically significant in all situations, rejecting the null hypothesis that the means are equal.

**Figure 2:**
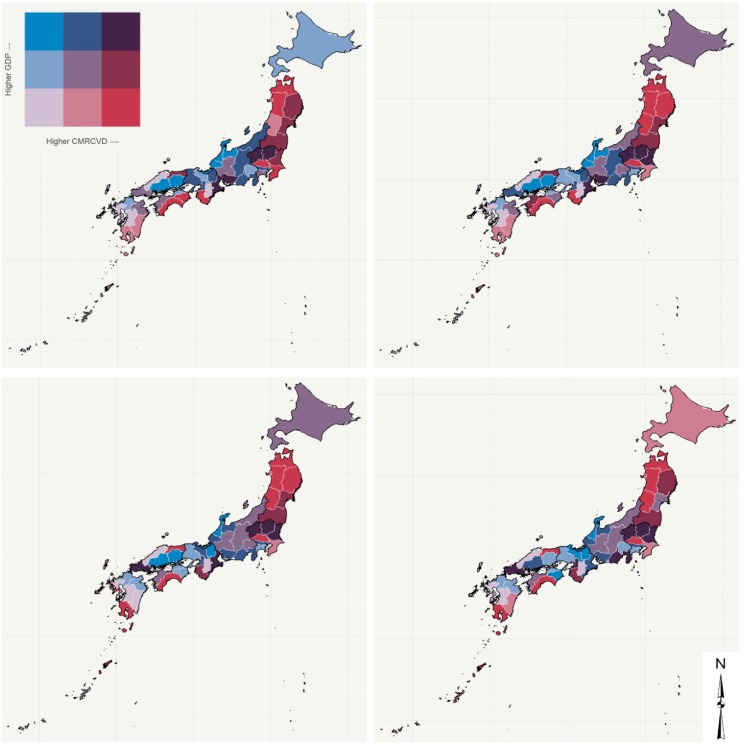
CVD mortality rates vs. GDP per capita, males, prefectures, Japan—time series 1996–2000 (**top left**), 2001–2005 (**top right**), 2006–2010 (**bottom left**), and 2011–2015 (**bottom right**).

**Figure 3:**
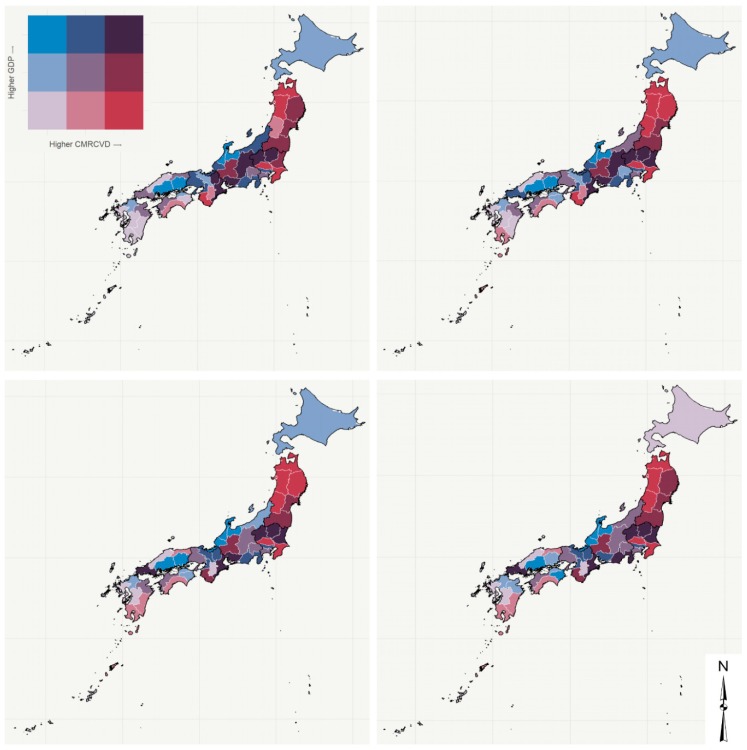
CVD mortality rates vs. GDP per capita, females, prefectures, Japan—time series 1996–2000 (**top left**), 2001–2005 (**top right**), 2006–2010 (**bottom left**), and 2011–2015 (**bottom right**).

**Figure 4:**
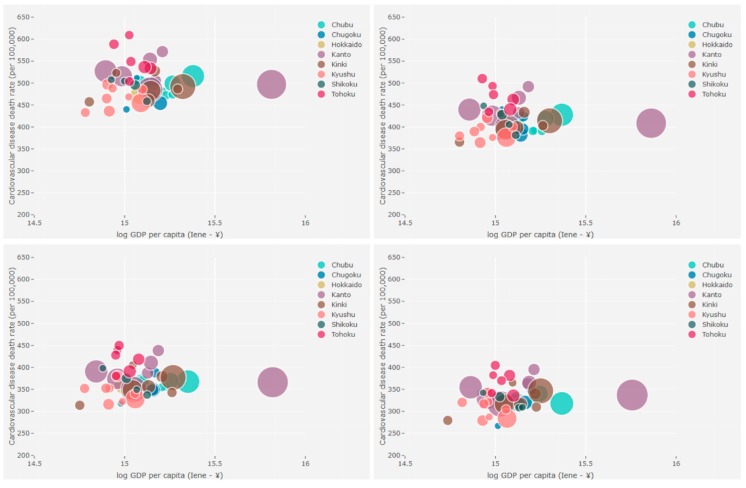
CVD mortality rates vs. GDP per capita vs. population, males, prefectures, Japan—time series 1996–2000 (**top left**), 2001–2005 (**top right**), 2006–2010 (**bottom left**), and 2011–2015 (**bottom right**).

**Figure 5:**
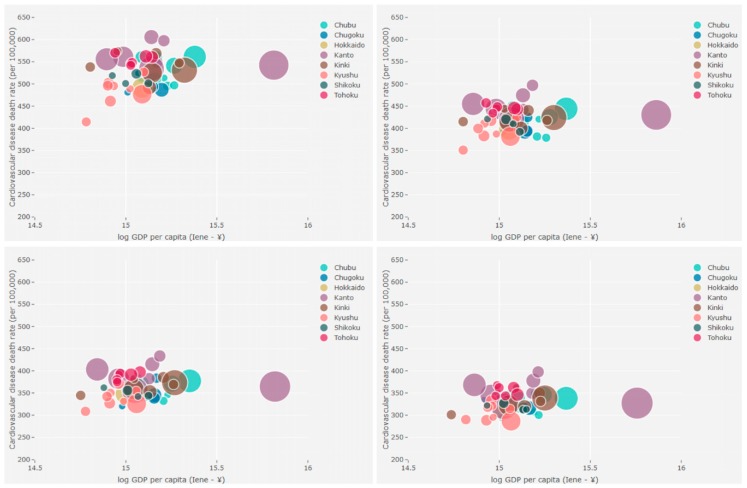
CVD mortality rates vs. GDP per capita vs. population, females, prefectures, Japan—time series 1996–2000 (**top left**), 2001–2005 (**top right**), 2006–2010 (**bottom left**), and 2011–2015 (**bottom right**).

Figure 2 (males) and Figure 3 (females) show the relationship between CVD mortality rates and GDP per capita in the four time series studied by prefecture through bivariate maps. The bubble charts presented in Figure 4 (males) and Figure 5 (females) corroborate the bivariate maps and bring a third variable (population), bubble size represents population size, that is helpful in understanding this relationship over time. The colors of the bubbles define the prefectures according to the geographic region in which they are located (Hokkaido, Tohoku, Kanto, Chubu, Kinki, Chugoku, Shikoku, and Kyushu).

The overall results show a decline in age-standardized CVD mortality rates in all prefectures over the period for both men and women. The results also suggest that GDP per capita has varied between 1996 and 2015. In general, the prefectures observed a fall in GDP per capita in the first 15 years of the period, then an increase between 2011 and 2015. Only Wakayama and Yamaguchi prefectures have experienced growth over the entire period. On the other hand, the prefectures of Chiba, Kanagawa, Ishikawa, Gifu, Shiga, Osaka, Nara, and Tottori showed a decline in GDP per capita throughout the period analyzed. In summary, the relationship between CVD mortality rates and GDP per capita at the prefecture level does not have an overall pattern of the same (high-high or low-low) or inverse association (high-low or low-high), but is instead a heterogeneous relationship. Even so, we would characterize Figure 2 and Figure 3 as showing a shift from stronger shades (purple, brown, red, etc.) to softer shades (light gray, light blue, light red, etc.) in the northeast–southwest direction of the country (Hokkaido is a particular case). We can also point out that although the CVD mortality rates in the northeast are higher than in the southwest, there is a similar level of GDP per capita during the study period. For example, GDP per capita in Tohoku and Kyushu are similar, but Tohoku has higher CVD mortality rates than Kyushu between 1996 and 2015 (Figure 4 and Figure 5).

More specifically, we would like to highlight certain prefectures/regions. In the Hokkaido region, a special case whose only prefecture is Hokkaido, GDP per capita in the initial three time series was in the second quantile (medium) and shifted to the first (low) between 2011 and 2015. Meanwhile, the age-standardized crude mortality rate from cardiovascular disease (CMRCVD) varied between the first (low) and second quantiles (medium) for men and remained constant (first quantile) for women.

The prefectures of Tohoku (Aomori, Akita, Iwate, Miyagi, and Fukushima) and Kanto (Tochigi, Ibaraki, and Saitama) regions are those with the highest CMRCVD. Of these, Saitama still stands out for having the third lowest GDP per capita and, at the other extreme, Tochigi has the ninth highest GDP per capita among the prefectures. In short, in the Tohoku region, the relationship between CMRCVD and GDP per capita varied mostly between brown (medium GDP per capita and high CMRCVD) and dark red (low GDP per capita and high CMRCVD), while in the Kanto region this relationship is heterogeneous. Tokyo, for example, is an outlier within the region, as it is the prefecture with the highest GDP per capita in the country, while its CMRCVD remained in the second quantile (medium) throughout the period.

At the other extreme, the prefectures of Kyushu region (Okinawa, Kumamoto, Nagasaki, Fukuoka, and Saga), as well as Shimane (Chugoku region) and Nara (Kinki region) prefectures, are those with the lowest CMRCVD. Okinawa, Kumamoto, Nagasaki, and Nara also stand out for having some of the lowest GDPs per capita among Japan’s prefectures. Cockerham et al. [32], when examining the social gradient theory of health and life expectancy in Okinawa, a prefecture and a southern peripheral island of Japan, found that Okinawans traditionally rank at the top in health and life expectancy (also driven by low CVD mortality rates) and at the bottom in socioeconomic indicators, like GDP. It has been persistently proven that GDP per capita is a powerful predictor of life expectancy at birth [33,34], which makes these four prefectures, in particular, anomalies that should be analyzed in future studies.

Finally, we would like to highlight, besides Tokyo, the other prefectures with the highest GDP per capita in the country. In general, these prefectures are concentrated in the Chubu region (Aichi, Shizuoka, Toyama, Fukui, and Ishikawa) and, to a lesser extent, in the Kinki region (Osaka and Shiga), both in the central portion of the country. With regard to CMRCVD, the prefectures of the Chubu region are mostly concentrated in the low/medium quantiles, except for Aichi, which is in the high quantile. Meanwhile, Osaka and Shiga are in quantiles with medium CMRCVD (in the last two time series for males, Shiga prefecture shifted from medium to low CMRCVD).

## 4. Discussion

The high proportion of elderly people, the decline of non-communicable diseases, lifestyle modification, universal health insurance coverage, medical advancement, and high-income are characteristics of a Japan that is in an advanced stage of epidemiological transition and is a model for several other countries [18,35,36]. At the same time, the aging of the Japanese population has a substantial effect on disease structure, as the elderly require long-term care and exert pressure on health-care expenditure and use of resources due to multimorbidity [19].

Although Japan has many of the best health indicators in the world, the pace of epidemiological transitions varies substantially by region, which raises concerns about increasing health variations in the country and its prefectures [19]. Minagawa and Saito [37] study disability-free life expectancy (DFLE-65) above age 65 for regions in Japan and show that it is very closely related to socioeconomic conditions. They find that higher income per capita and wealth conditions are related to higher DFLE-65. They argue that that improvements in health conditions could be reduced by improving income levels and labor market conditions for the elderly across regions.

In this paper, our goal was to investigate the relationship between CVD mortality and economic development, measured by gross domestic product (GDP) per capita, in Japanese prefectures over a 20-year period (1996 to 2015), without pretending, however, to establish a causal relationship between them.

Gu et al. [38], studying Eastern and Southeastern Asia, show that higher GDP per capita is associated with lower mortality rates. In addition, they found that a higher level of GDP per capita is consistently associated with a lower CVD mortality rate and, after a certain level of GDP, this relation tends to become less pronounced. Japan has undergone many changes in recent decades, such as economic stagnation and a gradual increase in income inequality [18,19,39]. Therefore, despite the convergence that led to reduced mortality and disability from most major diseases, variation between prefectures is growing. We argue that investigating the association between GDP per capita and CVD mortality contributes to understanding the mortality dynamic in the country, following the discussion by [37] for DFLE-65.

There is also an important question of reverse causality. Suhrcke and Urban [40] show that increasing CVD morbidity might affect economic growth because it limits productivity and labor force participation. We do not have, in this paper, evidence to suggest the direction of causality. Our results indicate a decline in age-standardized CVD mortality rates in all prefectures over the period for both men and women. However, the reduction was not uniform across prefectures, which may suggest that there was an uneven health transition in Japan during this period [19]. The results also suggest that GDP per capita has varied between 1996 and 2015. In general, the prefectures had a fall in GDP per capita in the first 15 years of the period, then an increase between 2011 and 2015. Japan has had an economic recession in recent decades, which has increased income inequality and brought about greater health disparities [18,41]. In summary, the relationship between CVD mortality rates and GDP per capita at the prefecture level does not have an overall pattern of the same (high-high or low-low) or inverse association (high-low or low-high), but is instead a heterogeneous relationship. Even so, we would characterize Figure 2 and Figure 3 as showing a shift from stronger shades (purple, brown, red, etc.) to softer shades (light gray, light blue, light red, etc.) in the northeast–southwest direction of the country. This pattern is consistent with other studies that point out that there is a tendency of higher mortality (e.g., cardiovascular disease) in the northeast part of the country [18,42]. According to Ikeda et al. [18], “this geographical gradient might be attributable to differences in risk profiles such as a higher prevalence of hypertension and diabetes in the northeastern prefectures that are related to lifestyles, health-care resources, and socioeconomic status.”

Tomoike et al. [43] investigate the regional distribution of cardiovascular health service in Japan. They find a reasonable distribution of health care for CVD across prefectures that, in turn, show strong relations to population size and GDP. This result suggests that provision of health care is uniformly distributed in the country and this would have a positive impact on reducing mortality across regions. Nakaya and Dorling [44] show that income is closely related to mortality variations in Japan, but with a negative relationship for the elderly. They claim that higher income related to higher mortality might be reflecting the past health status of the elderly population and it is not capturing positive income effect. They also argue that there are important cultural differences across regions in Japan that might explain different levels of mortality. For instance, it is suggested that western prefectures give better status for women and this could reflect in better health outcomes independent from income level.

Our results indicate that CVD mortality decline in all prefectures of Japan from 1996–2000 to 2011–2015 and the same time trend is observed for males and females. CVD standardized mortality rates for females are higher than for males in several prefectures both in the earlier and later periods of analysis. Nakaya and Dorling [44] suggested that this might be related to the socioeconomic status of women in different areas of Japan. Despite the decline in mortality rates, there is also strong regional variations across regions. Regional disparities for CVD mortality are in line with what is observed in other studies. Suzuki et al. [45] find that social and regional inequality in premature adult mortality has increased in Japan from 1970 to 2005. The authors suggested that this increase is related to worse economic conditions both at the individual and regional level. Tanaka et al. [46] find, using microdata, that rising numbers of suicides are closely related to widening of mortality differentials across occupations.

Our results are also in line with other studies. Anand et al. [47], in establishing the relative prevalence of risk factors, atherosclerosis, and CVD among people of Aboriginal and European ancestry in Canada, found that Aboriginal people had a higher prevalence of CVD, atherosclerosis, obesity, and poverty compared with European Canadians. They state that there is a clear inverse relationship between higher incomes and lower rates of risk factors and CVD. This relationship we observe, for example, in Toyama, Ishikawa, and Hiroshima prefectures. On the other hand, Roth et al. [13], studying demographic and epidemiological drivers of global CVD, investigate the relationship between changes in CVD due to age-specific death rates and changes in GDP per capita in several countries. They point out that despite the increase in GDP per capita and a decline in the number of CVD deaths in many countries, the two variables had a significant correlation only within the category of upper-middle income countries. If we bring this categorization to the prefecture level, do we find the same correlation? This is a question that should be on the agenda. In another study, Roth et al. [2] analyzed global, regional, and national burdens of CVD between 1990 and 2015. The study showed that significant declines in CVD mortality rates occurred in all high-income regions, but has plateaued in recent years. An alarming finding in this study, however, is that trends in CVD mortality have plateaued and are no longer declining for high-income regions. In the same direction, Lopez and Adair [16], when assessing recent CVD mortality trends in high-income populations, state that there is recent evidence to suggest a slowdown in the pace of reduction of CVD mortality. Baptista and Queiroz [14] investigate the spatial pattern of deaths from CVD in Brazil and identify regions with high levels of mortality and how they have changed over the recent periods of time. They show temporal clockwise change in the concentration of regions with high levels of CVD mortality surrounded by high level mortality areas moving from the South to the Northeast. In Japan, Nomura et al. [19] analyze regional variations of disease burden in the 47 Japanese prefectures from 1990 to 2015. They state that some causes of death have patterns largely determined by geography (higher rates in the northeast, and lower in the central and southwest), such as CVD mortality. Additionally, they point out that while cerebrovascular and ischemic heart diseases remain the leading causes of death in the period, they showed substantial declines in age-standardized rates. In addition, the speed of mortality decline from these causes, and many others, has stabilized since 2005 for both men and women. They also find a very weak correlation between all-cause mortality level and per capita health expenditures indicating that other factors play an important role on the observed variation in mortality.

Our analyses are also subject to limitations. The main one is that this study does not incorporate other major risk factors associated with CVD mortality, such as education, diet, blood pressure, tobacco smoking, and others, although we argue that a good understanding of GDP per capita is fundamental as it is one of the most widely used socioeconomic predictors of mortality and health [24,25,26,27,28,29]. Examining studies with these biases, we also found that they support and corroborate the geographical variation presented in this paper. For example, some prefectures in the northeast have very high smoking rates, especially in Hokkaido, Aomori, Iwate, Fukushima, and Gunma in 2016 [48]. Uechi et al. [49] analyzed the variation of salt intake in the 47 prefectures of the country via urinary excretion. They found that sodium excretion was high in the northeast region. In the same direction, Tomonari et al. [50] evaluated the relationship between dietary salt intake, blood pressure, and stroke mortality in 12 regions of Japan. They show that the amount of salt intake was a significant factor in determining the regional distributions of both blood pressure and stroke mortality; higher values of all three were found in the northeast of the country. Fukuda et al. [51] argued that health care provision in Japan ranks among the best in the world. However, there was a recent rise in inequality in the access to health care provision across regions of Japan mostly explained by the geographic distribution of services. Their results suggest that most of differences in health status and mortality are linked to socioeconomic inequality across regions that increased after economic shocks in the 1990s and economic policies implemented after that [51]. Suzuki et al. [41] observed that compositional effects (age, sex, and occupation distribution) played a small role in determining mortality differentials across regions. They argue that economic, social, and cultural context might be more important in explaining observed differences. Kondo et al. [52] investigated the relation of economic recession and self-rated health status across socioeconomic groups and show that although poor health status has declined across all groups during the 1990s, there was an increase in the gap between lowest and highest groups.

Although our results indicate that the magnitude of a relative variation in GDP per capita in the prefectures over the period was not as significant, there is evidence of an increase in income inequality that has led to greater health disparities [18]. The results from previous research are in line with our results and indicate the importance of further studies on health inequalities across regions of Japan to better understand the roles of socioeconomic and cultural aspects to determine health and mortality variation. One must take into account that it is a relative variation in a high-income country, which may lead to mistaken conclusions about the greater or lesser importance of this variable in explaining changes and disparities in population health. Even so, this study, to our knowledge, is the first to provide a comprehensive vision of the relationship between CVD mortality and GDP per capita in Japanese prefectures. We argue that this study provides useful clues to policy makers for establishing effective measures for public health planning and the prevention of deaths from CVD. As demonstrated by this study, mapping of the CVD burden in Japan helps to clarify regional differences in life expectancy and health status across regions and identify prefectures where more targeted policy attention may be needed.

## 5. Conclusions

We used a bivariate choropleth map that is new to health studies to investigate the relationship between mortality from cardiovascular disease (CVD) and GDP per capita, by sex, in Japanese prefectures from 1996 to 2015. The overall results show a decline in age-standardized CVD mortality rates in all prefectures, for both men and women, and suggest that GDP per capita has varied over the period. We also observed that the relationship between CVD mortality rates and GDP per capita at the prefecture level does not have an overall pattern of the same or inverse association, but is instead a heterogeneous relationship.

Our results and analysis answer a series of important research questions. We believe that incorporate other major risk factors associated with CVD mortality, such as education, healthcare access, lifestyle, environment, and others, is important in order to identify the contribution of each one of them to the mortality recorded in the prefectures. In this sense, spatial regression models can be used to examine the spatiotemporal relationships between these variables.

## Figures and Tables

**Table 1:** Descriptive statistics—cardiovascular disease (CVD) mortality rates and gross domestic product (GDP) per capita, by sex and period.

	Region	Obs	(Mean; Std. Dev.) 1996–2000	(Mean; Std. Dev.) 2001–2005	(Mean; Std. Dev.) 2006–2010	(Mean; Std. Dev.) 2011–2015	t-Statistic	*p*-Value	95% CI
Crude mortality rate from cardiovascular disease (CMRCVD), Males	Hokkaido	1	474.98; NA	405.75; NA	361.77; NA	323.34; NA			
	Tohoku	6	553.38; 38.80	469.05; 29.58	417.72; 27.17	369.21; 26.28			
	Kanto	7	522.14; 31.05	437.72; 32.35	388.10; 29.35	351.49; 26.21			
	Chubu	9	491.94; 15.14	408.23; 14.80	360.97; 8.39	323.44; 9.64			
	Kinki	7	489.94; 27.61	405.59; 23.26	360.31; 29.62	324.72; 28.10			
	Chugoku	5	474.98; 30.40	404.07; 27.11	356.34; 27.62	319.19; 32.51			
	Shikoku	4	491.57; 22.52	415.69; 28.70	364.80; 27.21	323.15; 17.16			
	Kyushu	8	465.92; 23.78	388.38; 18.15	342.84; 20.69	306.90; 22.13			
	Overall	47	497.36; 37.22	416.75; 33.54	368.92; 32.05	330.36; 29.38			
	1996–2000 vs. 2011–2015						46.11	2.47 × 10^−5^ ***	[156.94–173.91]
	2001–2005 vs. 2011–2015						37.83	7.91 × 10^−6^ ***	[81.21–92.04]
	2006–2010 vs. 2011–2015						25.55	1.63 × 10^−6^ ***	[35.32–42.53]
Crude mortality rate from cardiovascular disease (CMRCVD), Females	Hokkaido	1	491.44; NA	396.91; NA	348.19; NA	313.01; NA			
	Tohoku	6	554.84; 11.11	445.27; 7.57	388.09; 8.72	354.28; 11.03			
	Kanto	7	563.71; 27.40	451.81; 26.40	390.99; 27.68	355.13; 27.80			
	Chubu	9	528.05; 27.66	416.58; 24.81	358.28; 18.34	325.33; 17.01			
	Kinki	7	539.47; 27.84	422.29; 15.35	366.48; 15.05	331.99; 20.20			
	Chugoku	5	497.84; 16.79	403.62; 15.93	352.20; 25.89	321.87; 20.46			
	Shikoku	4	511.08; 11.44	410.81; 13.49	350.94; 9.57	318.57; 7.02			
	Kyushu	8	482.74; 33.49	394.30; 23.89	338.93; 19.89	305.78; 17.80			
	Overall	47	525.33; 36.86	420.26; 27.55	363.40; 25.67	329.92; 24.70			
	1996–2000 vs. 2011–2015						39.10	0.00025 ***	[181.24–204.57]
	2001–2005 vs. 2011–2015						53.39	1.01 × 10^−5^ ***	[85.49–93.42]
	2006–2010 vs. 2011–2015						54.45	1.12 × 10^−7^ ***	[32.06–4.97]
Gross domestic product (GDP) per capita	Hokkaido	1	3,576,523.69; NA	3,445,865.77; NA	3,262,695.18; NA	3,325,173.73; NA			
	Tohoku	6	3,433,025.80; 245,683.36	3,300,966.69; 226,786.18	3,239,177.83; 171,658.33	3,371,702.00; 170,078.19			
	Kanto	7	4,136,858.70; 1,476,231.68	4,092,849.10; 1,650,936.63	4,030,843.22; 1,544,894.14	4,028,397.73; 1,374,579.74			
	Chubu	9	4,027,838.09; 383,857.03	3,984,126.60; 399,028.09	3,897,409.85; 385,307.15	3,904,454.85; 383,350.29			
	Kinki	7	3,722,355.37; 646,676.02	3,668,318.87; 581,919.85	3,664,780.70; 606,194.88	3,668,725.81; 582,640.94			
	Chugoku	5	3,657,794.16; 263,055.59	3,620,914.78; 239,808.56	3,577,668.03; 354,972.07	3,579,774.87; 408,422.44			
	Shikoku	4	3,364,701.83; 282,630.10	3,410,899.08; 255,786.70	3,348,105.72; 343,002.51	3,482,203.85; 344,634.99			
	Kyushu	8	3,143,210.18; 336,695.29	3,127,890.12; 299,193.59	3,106,894.10; 282,077.80	3,152,639.24; 243,958.09			
	Overall	47	3,666,663.39; 722,486.00	3,621,452.60; 758,963.03	3,569,781.58; 729,090.58	3,609,024.42; 664,783.23			
	1996–2000 vs. 2011–2015						1.83	0.00031 ***	[−20,202.24–157,511.17]
	2001–2005 vs. 2011–2015						0.69	1.82 × 10^−5^ ***	[−41,816.68–76,506.40]
	2006–2010 vs. 2011–2015						−2.38	2.48 × 10^−5^ ***	[−96,151.28–−223.09]

Signif. codes: * *p* ≤ 0.05; ** *p* ≤ 0.01; *** *p* ≤ 0.001.
